# Supplementary material for: Ibuprofen-Loaded Heparin Modified Thermosensitive Hydrogel for Inhibiting Excessive Inflammation and Promoting Wound Healing
Source: Polymers (Basel). 2020 Nov 6;12(11):2619. doi: 10.3390/polym12112619 (PMC7694755; doi:10.3390/polym12112619)
Supplement: Supplementary file 1 [file polymers-12-02619-s001.pdf]

Suportive Infoemation

Ibuprofen-Loaded Heparin Modified Thermosensitive Hydrogel for Inhibiting Excessive Inflammation and Promoting Wound Healing

Abegaz Tizazu Andrgie [1], Haile Fentahun Darge [1], Tefera Worku Mekonnen [1], Yihenew Simegniew Birhan [1], Endiries Yibru Hanurry [1], Hsiao-Ying Chou [1], Chih-Feng Wang [1,2,] Hsieh-Chih Tsai [1,2,*], Jen Ming Yang [3,4,*] and Yen-Hsiang Chang [3,4]

[1] Graduate Institute of Applied Science and Technology, National Taiwan University of Science and Technology, Taipei 106, Taiwan; habegaz21@gmail.com (A.T.A.); fentahunhailebdu@gmail.com (H.F.D.); tefe16@gmail.com (T.W.M.); yihenews@gmail.com (Y.S.B.); Endris_Yibru@dmu.edu.et (E.Y.H.); wherelove8@gmail.com (H.-Y.C.); cfwang@mail.ntust.edu.tw (C.-F.W.)

[2] Advanced Membrane Materials Center, National Taiwan University of Science and Technology, Taipei 106, Taiwan

[3] Department of Chemical and Materials Engineering, Chang Gung University, Taoyuan 320-338, Taiwan; cyh4d25@adm.cgmh.org.tw

[4] Department of General Dentistry, Chang Gung Memorial Hospital, Taoyuan 320-338, Taiwan

* Correspondence: h.c.tsai@mail.ntust.edu.tw (H.-C.T.); jmyang@mail.cgu.edu.tw (J.M.Y.)

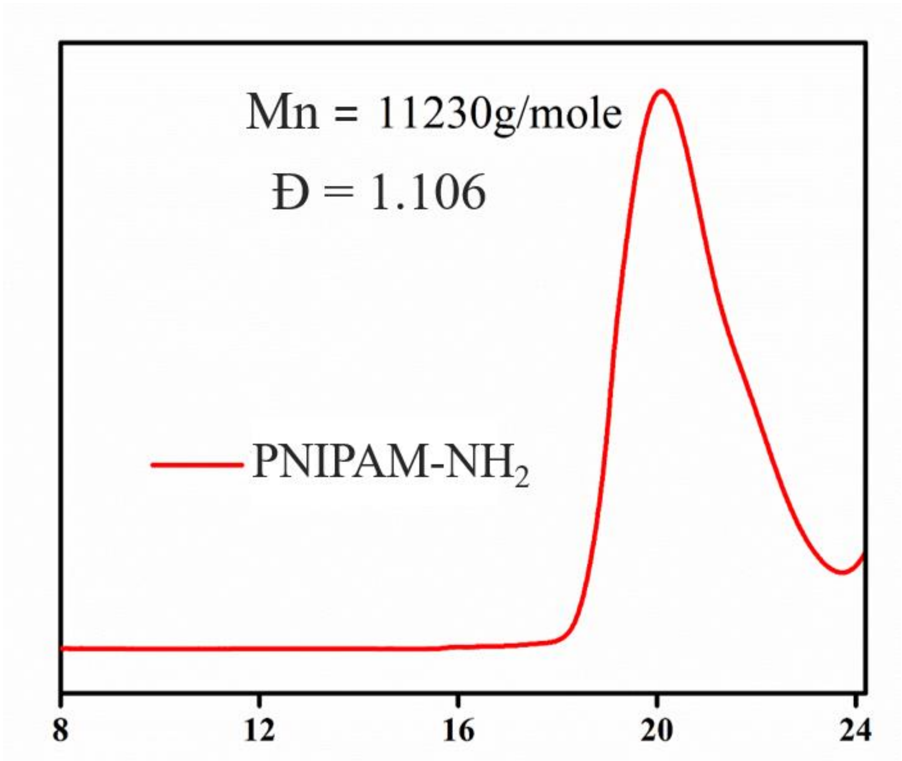

Figure S1. Number average molecular weight (Mn) of PNIPAM-NH_2 from GPC measurement.

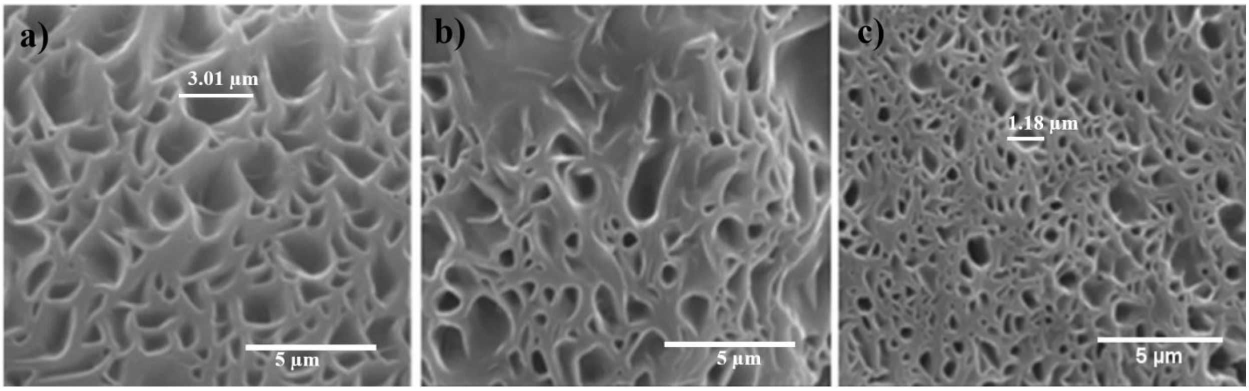

Figure S2. SEM images of lyophilized (a) Hep-PNIPAM-b_1 hydrogel and (b) Hep-PNIPAM-b_2 hydrogel, and (C) Hep-PNIPAM-b_3 hydrogel.

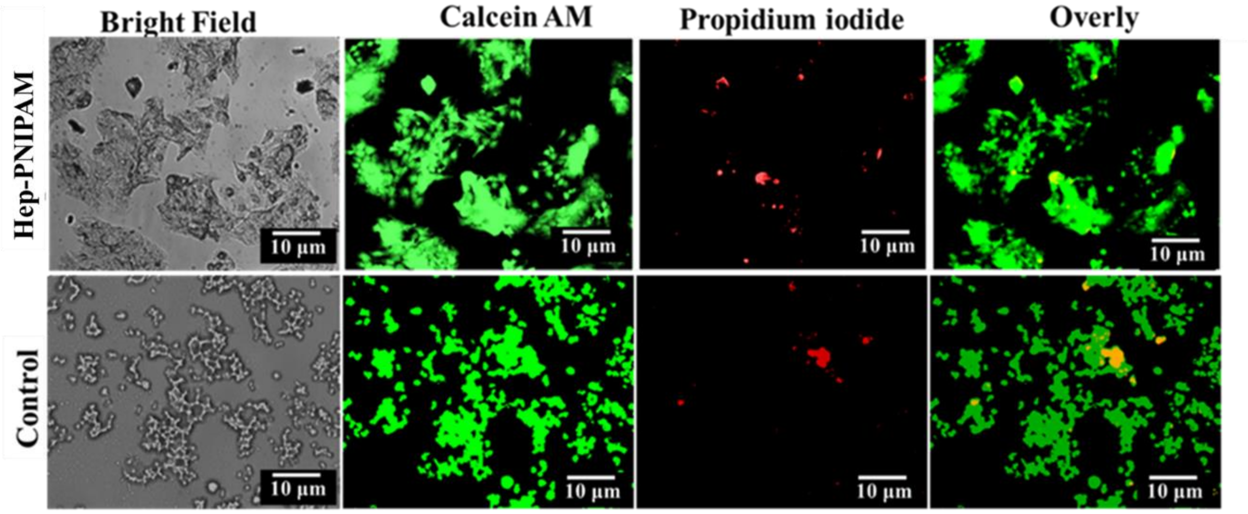

Figure S3. Fluorescence images of the HaCaT cells stained with calcein AM/PI with and without Hep-PNIPAM treatment.

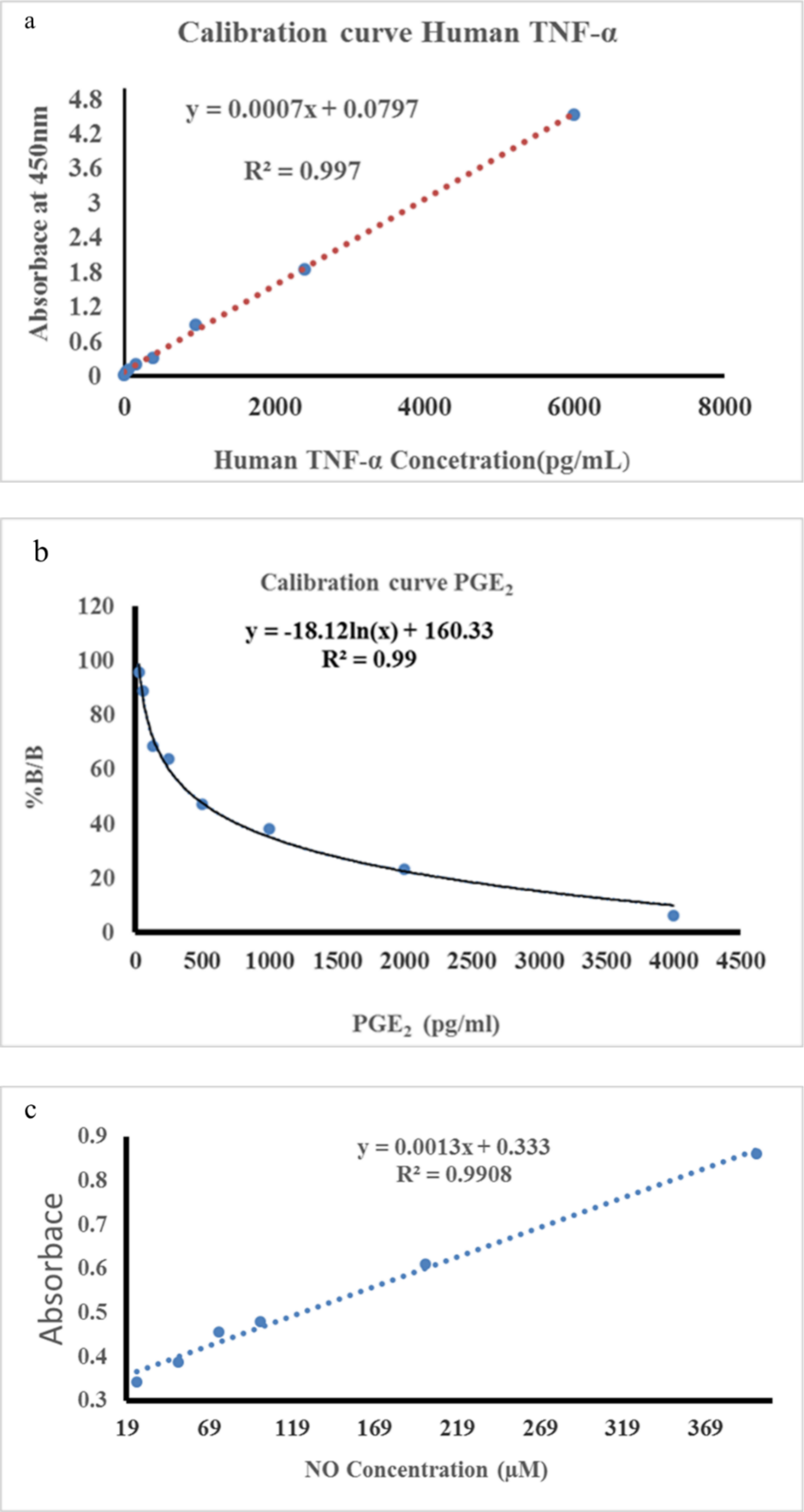

Figure S4. The standard calibration curve of (a) TNF-α, (b). PGE_2 and(c) NO.
